# Aaptamines from the Marine Sponge *Aaptos* sp. Display Anticancer Activities in Human Cancer Cell Lines and Modulate AP-1-, NF-*κ*B-, and p53-Dependent Transcriptional Activity in Mouse JB6 Cl41 Cells

**DOI:** 10.1155/2014/469309

**Published:** 2014-07-23

**Authors:** Sergey A. Dyshlovoy, Sergey N. Fedorov, Larisa K. Shubina, Alexandra S. Kuzmich, Carsten Bokemeyer, Gunhild Keller-von Amsberg, Friedemann Honecker

**Affiliations:** ^1^Laboratory of Marine Natural Products Chemistry, G.B. Elyakov Pacific Institute of Bioorganic Chemistry, Far-East Branch of the Russian Academy of Sciences, Prospect 100 Let Vladivostoku 159, Vladivostok 690022, Russia; ^2^Department of Oncology, Haematology and Bone Marrow Transplantation with Section Pneumology, Hubertus Wald-Tumorzentrum, University Medical Center Hamburg-Eppendorf, Martinistraße 52, 20246 Hamburg, Germany; ^3^School of Natural Sciences, Far East Federal University, Sukhanova Street 8, Vladivostok 690950, Russia; ^4^Tumor and Breast Center ZeTuP St. Gallen, Rorschacherstraße 150, 9006 St. Gallen, Switzerland

## Abstract

Aaptamine (8,9-dimethoxy-1H-benzo[de][1,6]naphthyridine) is a marine natural compound possessing antioxidative, antimicrobial, antifungal, and antiretroviral activity. Earlier, we have found that aaptamine and its derivatives demonstrate equal anticancer effects against the human germ cell cancer cell lines NT2 and NT2-R and cause some changes in the proteome of these cells. In order to explore further the mechanism of action of aaptamine and its derivatives, we studied the effects of aaptamine (**1**), demethyl(oxy)aaptamine (**2**), and isoaaptamine (**3**) on human cancer cell lines and on AP-1-, NF-*κ*B-, and p53-dependent transcriptional activity in murine JB6 Cl41 cells. We showed that compounds** 1**–**3** demonstrate anticancer activity in THP-1, HeLa, SNU-C4, SK-MEL-28, and MDA-MB-231 human cancer cell lines. Additionally, all compounds were found to prevent EGF-induced neoplastic transformation of murine JB6 Cl41 cells. Nuclear factors AP-1, NF-*κ*B, and p53 are involved in the cellular response to high and nontoxic concentrations of aaptamine alkaloids** 1**–**3**. Furthermore, inhibition of EGF-induced JB6 cell transformation, which is exerted by the compounds** 1**–**3** at low nontoxic concentrations of 0.7–2.1 *μ*M, cannot be explained by activation of AP-1 and NF-*κ*B.

## 1. Introduction

**Figure 1:**
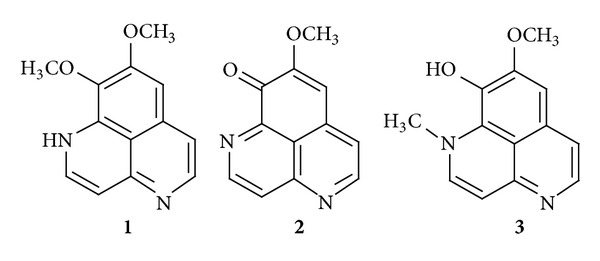
Structures of aaptamine (**1**), demethyl(oxy)aaptamine (**2**), and isoaaptamine (**3**).

Aaptamines are a group of bioactive benzo[*de*][1,6]-naphthyridine alkaloids, initially isolated from marine sponges mostly belonging to the genus* Aaptos*. These compounds have been found to possess a variety of biological activities (for review see [1]). Among those, an anticancer effect has been the most frequently reported for aaptamines, although the underlying mechanism is poorly understood. Aaptamine has been shown to have DNA intercalating activity [2] as well as the ability to induce a p21-mediated (but p53-independent) G2/M cell cycle arrest [3–5]. Our recently reported global proteome screen of proteins differentially regulated upon treatment with aaptamine (**1**) and its derivatives** 2**,** 3** in the human embryonal carcinoma cell lines NT2 and NT2-R, a cisplatin-resistant subline, uncovered several possible protein targets of these alkaloids [5, 6]. In the present study, the effects of the alkaloids** 1**–**3** (Figure 1) on AP-1, NF-*κ*B, and p53 transcriptional activity were investigated in order to further explore the mechanism of action of these compounds [7]. As a result, we present a more detailed picture of the biological action of aaptamines, extending the knowledge gained from previous research [5–11].

## 2. Materials and Methods

### 2.1. Reagents

Aaptamine compounds** 1**–**3** were isolated from the marine sponge* Aaptos* sp. as described before [7, 8]. Epidermal growth factor (EGF) was purchased from Collaborative Research (Bedford, MA, USA), the Cell Titer 96 Aqueous One Solution Reagent [3-(4,5-dimethylthiazol-2-yl)-5-(3-carboxymethoxyphenyl)-2-(4-sulfophenyl)-2H-tetrazolium, inner salt (MTS)] Kit was purchased from Promega (Madison, WI, USA), and D-luciferin was purchased from Anaspec (Waddinxveen, The Netherlands).

### 2.2. Cell Culture

The murine epidermal cell line JB6 P^+^ Cl41 and its stable transfectants JB6-Luc AP-1, JB6-Luc NF-*κ*B, or JB6-Luc p53 (PG-13) cells were cultured at 37°C and 5% CO_2_ in MEM, containing 5% FBS, 2 mM L-glutamine, and 1% penicillin/streptomycin (Invitrogen, Paisley, UK). The human cancer cell lines HeLa (cervical carcinoma), SNU-C4 (colon cancer), and THP-1 (monocytic leukemia) were cultured at 37°C and 5% CO_2_ in RPMI medium containing 10% FBS, 2 mM L-glutamine, and 1% penicillin/streptomycin. The human cancer cell lines MDA-MB-231 (breast cancer) and SK-MEL-28 (melanoma) were cultured at 37°C and 5% CO_2_ in DMEM medium containing 10% FBS, 2 mM L-glutamine, and 1% penicillin/streptomycin. The THP-1 cell line was cultured in suspension; other cell lines were cultured in monolayers. JB6 cell lines were kindly provided by Dr. Zigang Dong, Hormel Institute, University of Minnesota, MN, USA. The cancer cell lines were purchased from the ATCC collection. Information regarding the genetic background of these cell lines is available online at the ATCC website.

### 2.3. Cytotoxicity Assay (MTS Test)

The effect of the substances on cell viability was evaluated using the MTS test [12]. The cells were preincubated overnight in 96-well plates (6 × 10^3^ per well), 100 *μ*L/well for adherent cells, or 50 *μ*L/well for suspension (THP-1 cells). Then, the medium was replaced with fresh medium containing the substances at various concentrations in a total volume of 100 *μ*L/well for adherent cells, and for suspension cells, 50 *μ*L/well of fresh substance-containing medium was added and the cells were incubated for 24 h. Then 20 *μ*L of Cell Titer 96 Aqueous One Solution Reagent was added into each well, and MTS reduction was measured 2 h later spectrophotometrically at 492 and 690 nm as background using *μ*Quant equipment (Bio-Tek Instruments, Winooski, VT, USA). Results are represented as IC_50_ of the substances against corresponding untreated cells.

### 2.4. Anchorage-Independent Neoplastic Transformation Assay

The cancer preventive effect of aaptamine derivatives was evaluated using an anchorage-independent neoplastic transformation assay, as described previously [13]. EGF (10 ng/mL) was used to induce neoplastic transformation of JB6 P^+^ Cl41 cells. JB6 P^+^ Cl41 cells (8 × 10^3^ cells/mL) were treated in 6-well plates with various concentrations of the substances in 1 mL of 0.33% basal medium Eagle- (BME-) agar containing 10% FBS over 3 mL of 0.5% BME-agar containing 10% FBS and various concentrations of the substances. The plates were incubated at 37°C in a humidified atmosphere with 5% CO_2_ for 1 week, before cell colonies were scored using an Olympus CKX31 inverted research microscope (Olympus, Center Valley, PA, USA). The ability of the substances to inhibit neoplastic transformation of JB6 P^+^ Cl41 cells is represented as concentration-dependent correlation of number of cell colonies.

### 2.5. Determination of the Effect of the Substances on the Basal Transcriptional Activity of AP-1, NF-*κ*B, or p53 Nuclear Factors

The effects of the substances on the basal transcriptional activities of AP-1, NF-*κ*B, or p53 nuclear factor were evaluated using JB6 Cl41 cell lines stably expressing a luciferase reporter gene controlled by an AP-1-, NF-*κ*B-, or p53-DNA binding sequence, as described previously [14]. Briefly, cells were preincubated overnight in 96-well plates (20 × 10^3^ cells/well) in 100 *μ*L/well of culture medium. Then the medium was replaced with fresh medium containing different concentrations of the substances tested. After incubation for 6 h or 24 h, cell viability and the effect on the transcriptional activities of the nuclear factors were determined. To determine the transcriptional activities, cells were lysed for 1 h at RT with lysis buffer (0.1 M PBS (pH 7.8), 1% Triton X-100, 1 mM DTT, 2 mM EDTA). Then, 50 *μ*L of lysate from each well was transferred into a luminescent analysis plate, and luciferase activity was measured using luciferase assay buffer (100 *μ*L/well) (0.47 mM D-luciferin, 20 mM Tricin, 1.07 mM (MgCO_3_)_4_ × Mg(OH)_2_ × 5H_2_O, 2.67 mM MgSO_4_ × 7H_2_O, 33.3 mM DTT, 0.53 mM ATP, 0.27 mM CoA, and 0.1 mM EDTA (pH 7.8)) and the Luminoscan Ascent Type 392 microplate reader (Labsystems, Helsinki, Finland). The results are presented as a concentration-dependent correlation of transcriptional activity of AP-1, NF-*κ*B, or p53 nuclear factors.

### 2.6. Apoptosis Assay

The onset of early and late apoptosis was analyzed by flow cytometry using Annexin V-FITC and propidium iodide (PI) double staining. 1 × 10^6^ cells per 10 cm dish containing 10% FBS-RPMI were treated with various concentrations of substances** 1**–**3** for 24 hours. After incubation, cells were washed with PBS by centrifugation at 1000 rpm for 5 min and processed for detection of apoptosis using Annexin V-FITC and PI staining according to the manufacturer's protocol. In brief, 1 × 10^5^–5 × 10^5^ cells were resuspended in 500 *μ*L of 1× binding buffer (Annexin V-FITC Apoptosis Detection Kit). Then, 5 *μ*L of Annexin V-FITC and 5 *μ*L of PI were added, and the cells were incubated at room temperature for 15 min in the dark and were analyzed by flow cytometry.

## 3. Results

### 3.1. Aaptamines Alter AP-1-, NF-*κ*B-, and p53-Dependent Transcriptional Activity in JB6 Cl41 Cells

**Figure 2:**
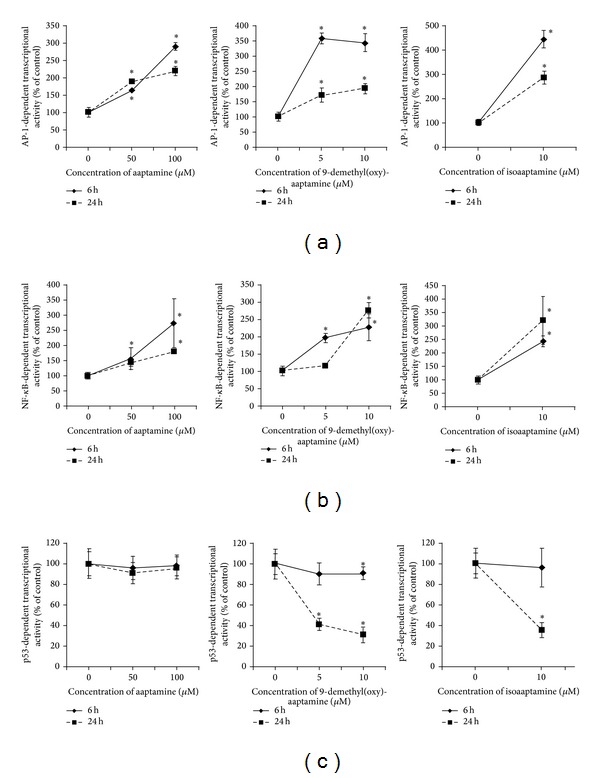
Effects of aaptamines** 1**–**3** on the transcriptional activity of AP-1 (a), NF-*κ*B (b), or p53 (c) in JB6 Cl41 cells stably expressing a luciferase reporter gene controlled by AP-1, NF-*κ*B, or p53 DNA binding sequences, respectively. Cells were treated with the indicated concentrations of the substances for 6 h or 24 h. All experiments were performed in triplicate and repeated at least two times. ^*“*∗*”*^-*P* < 0.05, statistically significant differences between treated and untreated control cells (Student's *t*-test).

The effects of aaptamines** 1**–**3** on the transcriptional activities of the nuclear factors AP-1, NF-*κ*B, and p53 were examined by using the luciferase assay and JB6 Cl41 cells stably expressing a luciferase reporter gene controlled by AP-1, NF-*κ*B, or p53 DNA binding sequences. The short-term effect of aaptamines on the transcriptional activity after 6 h of treatment and the long-term effect after 24 h of treatment were examined. Results are shown in Figure 2 as concentration-dependent correlations of transcriptional activities (as percentage of untreated control cells). Unexpectedly, we found that aaptamine alkaloids** 1**–**3** consistantly activate AP-1- and NF-*κ*B-dependent transcriptional activity at nontoxic concentrations after both 6 h and 24 h of treatment (Figure 2). The observed effects were similar to those for cisplatin (data not shown), a widely used anticancer cytotoxic drug, which also activates AP-1- and NF-*κ*B-dependent transcriptional activity at noncytotoxic concentration in JB6 Cl41 cells.

Neither aaptamine (**1**) nor demethyl(oxy)aaptamine (**2**) or isoaaptamine (**3**) activated p53-dependent transcriptional activity. Moreover, derivatives** 2 **and** 3** as well as cisplatin downregulated p53 transcriptional activity at noncytotoxic concentrations. In fact, this finding is in line with previous observations suggesting p53-independent cell cycle arrest in aaptamine treated cells at noncytotoxic concentrations of the drug [3–5]. Therefore, we speculate that apoptosis induced by demethyl(oxy)aaptamine (**2**) and isoaaptamine (**3**) [8] is not p53-dependent, similar to the situation reported after treatment of fibroblasts with cisplatin [15], although additional experiments are necessary to confirm this assumption.

In addition, we carried out similar experiments using a number of significantly lower concentrations (0.25; 0.5; 1.0; 2.0 *μ*M) of the substances** 1**–**3**. Neither aaptamine (**1**) nor demethyl(oxy)aaptamine (**2**) or isoaaptamine (**3**) affects AP-1, NF-*κ*B, or p53-dependent transcriptional activities in this range of low, nontoxic concentrations.

### 3.2. Analysis of Anticancer Activity of Aaptamines

The effects of the aaptamines** 1**–**3** on the viability of five human cancer cell lines were studied using the MTS assay. It was shown that demethyl(oxy)aaptamine (**2**) and isoaaptamine (**3**) demonstrate higher anticancer activity than the mother compound aaptamine (**1**) (Table 1).

### 3.3. Aaptamines Induce Apoptosis in THP-1 Human Leukemia Cells

**Figure 3:**
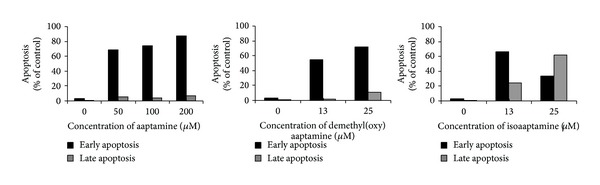
The induction of apoptosis by aaptamines** 1**–**3** in THP-1 human cancer cells. Cells were treated with the indicated concentrations of the substances for 24 h.

To study whether aaptamine alkaloids** 1**–**3** induce apoptosis in human cancer cells, we analysed THP-1 cells by flow cytometry after treatment with the compounds. The results show that aaptamine, demethyl(oxy)aaptamine, and isoaaptamine induce apoptosis in THP-1 cells in a dose-dependent manner (Figure 3). In these experiments, aaptamine (**1**) was also less active than the other two alkaloids** 2** and** 3**.

### 3.4. Aaptamines Prevent EGF-Induced Transformation of JB6 P^+^ Cl41 Cells

The ability of the aaptamine alkaloids** 1**–**3** to prevent EGF-induced neoplastic transformation and colony formation of murine epithelial JB6 P^+^ Cl41 cells was studied using anchorage independent soft agar assay.

**Figure 4:**
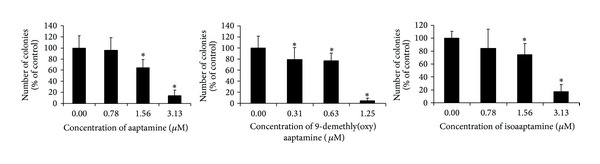
Effects of aaptamines** 1**–**3** on the EGF-induced neoplastic transformation and colony formation of murine epithelial JB6 P^+^ Cl41 cells. Cells in a soft agar were treated with the indicated concentrations of the substances for one week. All experiments were performed in triplicate and repeated at least two times. ^*“*∗*”*^-*P* < 0.05, statistically significant differences between treated and untreated control cells (Student's *t*-test).

This clone of JB6 cells is sensitive (P^+^) to tumor promoters like EGF or 12-O-tetradecanoylphorbol-13-acetate (TPA) and shows neoplastic transformation and anchorage independent colony formation upon stimulation with these agents. As shown in Figure 4, the substances examined were able to inhibit EGF-induced neoplastic transformation and colony formation of JB6 P^+^ Cl41 cells at low, noncytotoxic concentrations. Demethyl(oxy)aaptamine (**2**) possessed the strongest activity in preventing colony formation, INCC_50_ = 0.7 *μ*M, whereas both aaptamine and isoaaptamine showed INCC_50_ = 2.1 *μ*M (Figure 4).

## 4. Discussion

The transcription factors activator protein-1 (AP-1) and nuclear factor kappa B (NF-*κ*B) are strongly involved in regulation of a wide range of cellular processes, including cell migration, proliferation, differentiation, inflammation, survival, and immunity [16–22].

Both nuclear factors are implicated not only in cell transformation and tumor promotion, but also in the induction of apoptosis and tumor suppression [16, 21, 23–33]. Some of the AP-1 proteins, such as Jun-B and c-Fos, were shown to have tumor-suppressor activity both* in vitro* and* in vivo* [34, 35]. Activation of another AP-1 protein, c-Jun, is required for the induction of Fas L-mediated apoptosis in PC12 and human leukemia HL-60 cells [36, 37]. Transactivated AP-1 protein inhibits proliferation of activated T cells [38]. Activation of both AP-1 and NF-*κ*B nuclear factors is necessary for apoptosis by DNA damaging agents and ceramide in T lymphocytes and Jurkat T cells [31, 32]. NF-*κ*B activation is required for apoptosis in fibrocystin/polyductin-depleted kidney epithelial cells [33]. One member of the AP-1 protein family, activating transcription factor 2 (ATF2), has tumor suppressor activities in nonmalignant skin tumors and breast cancer [39]. The balance between AP-1 family members, c-Jun, and ATF2 governs the choice between differentiation and apoptosis in PC12 cells [40]. The ultimate fate of the cells relies on the relative abundance of AP-1 or NF-*κ*B complexes, their compositions, cell type, and cellular environment [41]. It was reported that some cancer preventive and therapeutic compounds, as well as DNA damaging agents, including those of marine origin, can induce AP-1 and/or NF-*κ*B activities. For example, anticancer drug vinblastine, cancer preventive flavonoids kaempferol and genistein, anti-inflammatory drug tolfenamic acid, and marine alkaloids 3- and 10-bromofascaplysins all induce AP-1 activity [31, 42–46]. Marine compound 3-demethylubiquinone Q2 from ascidian* Aplidium glabrum* and its synthetic analogs, as well as the cancer preventive terpenoid dactylone, induce AP-1 and NF-*κ*B and at the same time inhibit p53-dependent transcriptional activities [47–50].

Our investigations demonstrate that aaptamine alkaloids** 1**–**3** induce AP-1 and NF-*κ*B- dependent transcriptional activity at high nontoxic concentrations (100% viable cells) (Figure 2). For aaptamine (**1**), such concentrations are 50–100 *μ*M; for 9-demethyl(oxy)aaptamine (**2**) -5–10 *μ*M; and for isoaaptamine (**3**) about 10 *μ*M. On the other hand, it was shown that neither aaptamine (**1**) nor demethyl(oxy)aaptamine (**2**) or isoaaptamine (**3**) affects the AP-1, NF-*κ*B, or p53-dependent transcriptional activity at low nontoxic concentrations of 0.25–2.0 *μ*M.

As was also demonstrated, aaptamines** 1**–**3** show inhibition of anchorage-independent EGF-induced JB6 cell transformation and colony formation in soft agar at low nontoxic concentrations (Figure 3) of 0.7–2.1 *μ*M. Therefore, inhibition of transformation of JB6 cells by aaptamines** 1**–**3** cannot be explained by the induction of AP-1 and NF-*κ*B-dependent transcriptional activity. Therefore, the molecular mechanisms underlying the cancer preventive effects of aaptamine and its derivatives at low nontoxic concentrations still remain unknown and await further investigations.

We showed that aaptamine (**1**) and its derivatives** 2, 3** demonstrate anticancer effects against five human tumor cell lines. The IC_50_ for aaptamine is about 150 *μ*M, and for alkaloids** 2**,** 3** from 10 to 70 *μ*M. At similar concentrations, these substances induced apoptosis in THP-1 human leukemia cells. Therefore, the anticancer effect of aaptamine, 9-demethyl(oxy)aaptamine, and isoaaptamine, can be at least in part explained by the induction of classical apoptosis.

## 5. Conclusions

Our study results indicate that the nuclear factors AP-1, NF-*κ*B, and p53 are involved in the cellular response following treatment with high nontoxic (but not with low nontoxic) concentrations of aaptamine alkaloids** 1**–**3**. It was also found that aaptamine (**1**) at high nontoxic concentrations exerts biological action independently of p53-dependent transcriptional activation, whereas aaptamine analogues** 2** and** 3** inhibited p53 activation. We also provide evidence for cancer preventive activity of all aaptamines, which is exerted at low nontoxic concentrations and therefore independently of AP-1 and NF-*κ*B activation.

## Figures and Tables

**Table 1:** Anticancer activity of aaptamines **1**–**3** against several human cancer cell lines. Cells were treated with the indicated concentrations of the substances for 24 h. All experiments were performed in triplicate.

Cancer type	Cell line	Compound (IC_50_, *μ*M)		
		**1**	**2**	**3**
Monocytic leukemia	THP-1	161.3 ± 20.2	40.9 ± 9.9	32.2 ± 6.8
Cervical carcinoma	HeLa	151.1 ± 10.8	18.6 ± 1.7	50.7 ± 3.6
Colon cancer	SNU-C4	267 ± 24.4	22.3 ± 6.9	35.8 ± 5.8
Melanoma	SK-MEL-28	156.5 ± 6.7	35.0 ± 2.2	70.3 ± 3.3
Breast cancer	MDA-MB-231	147.2 ± 3.9	9.1 ± 1.4	10.6 ± 2.8
